# COVID-19 and diffusing capacity of the lungs for carbon monoxide: a clinical biomarker in postacute care settings

**DOI:** 10.2217/bmm-2021-0134

**Published:** 2021-05-14

**Authors:** Salvatore Fuschillo, Pasquale Ambrosino, Andrea Motta, Mauro Maniscalco

**Affiliations:** ^1^Department of Pulmonary Rehabilitation, Istituti Clinici Scientifici Maugeri IRCCS, 27100, Pavia, Italy; ^2^Institute of Biomolecular Chemistry, National Research Council, 80078, Pozzuoli, Naples, Italy

**Keywords:** biomarkers, COVID-19, diffusing capacity of the lungs for carbon monoxide, DLCO, rehabilitation, spirometry

In December 2019, the novel SARS-CoV-2 was responsible for the COVID-19 outbreak in Wuhan, China [1]. Shortly after, the infection spread worldwide, and on 11 March 2020, the WHO declared COVID-19 a global pandemic.

COVID-19 is a multifaceted disease with respiratory, neurological, cardiovascular and digestive involvement, probably due to systemic endothelial dysfunction [2]. Although, the lung is the main organ affected by COVID-19, the clinical manifestations of the disease are widely unpredictable, ranging from no symptoms to severe respiratory impairment, leading in around 5% of the cases to intensive care unit admission due to acute respiratory failure and acute respiratory distress syndrome [3].

However, although a negative result from a swab test may extinguish the risk of contagion, it does not mark the end of the disease course. Reports of persistent symptoms in patients recovering from COVID-19 are emerging, thus suggesting the presence of a ‘post-COVID-19 syndrome’, even among patients who experienced a mild–acute illness [4]. In keeping with this, a number of studies have suggested that COVID-19 patients may not revert to baseline functional status and to baseline levels of healthcare needs after infection. Thus, given the complexity and variability of clinical manifestations and possible long-term outcomes, the implementation of postacute care strategies for COVID-19 patients may represent a step forward in the management of these patients after a negative swab test [5,6].

Persistent and long-lasting symptoms due to pulmonary function impairment and reduced exercise capacity have been previously reported in SARS and MERS. In patients who have recovered from SARS, a significant impairment in diffusing capacity of the lungs for carbon monoxide (DLCO) was documented in 27.3% [7], with this percentage found to be even higher (37%) for MERS patients at 1-year follow-up [8].

Similarly, after discharge from the acute care setting, COVID-19 patients may still have both residual computed tomography (CT) and functional abnormalities. The most common radiographic finding is represented by ground grass opacities, while a reduced DLCO with restrictive ventilator defects are common functional postacute sequelae. The severity and duration of both DLCO impairment and restrictive ventilator defects seem to be related to the severity of the acute disease. Overall, pulmonary function abnormalities may persist for up to 6 months or more, with the possibility that vascular and alveolar remodeling could evolve into pulmonary fibrosis in a number of patients [9].

In 110 post-COVID-19 patients with mild–severe disease, Mo *et al.* reported anomalies of DLCO percentage predicted in 47.2%, of total lung capacity (TLC) percentage predicted in 25%, of forced expiratory volume in 1 s (FEV1) percentage predicted in 13.6%, of forced vital capacity (FVC) percentage predicted in 9.1%, of FEV1/FVC in 4.5% and of small airway function in 7.3% of cases. The frequency of DLCO impairment paralleled the degree of pneumonia severity, being reported in 30.4% of mild, 42.4% of moderate and up to 84.2% of severe patients [10].

In a prospective study carried out 12 weeks after the onset of symptoms in previously hospitalized COVID-19 patients, abnormal DLCO was documented in 52%, with 45% of these having a concomitant restrictive ventilator defect. Interestingly, all patients presenting oxyhemoglobin desaturation with exercise had abnormal DLCO. A strong association was documented between the number of days spent on oxygen supplementation during the acute phase and both DLCO percentage predicted and total CT score. Similarly, a strong association of dyspnea severity with DLCO percentage predicted was observed [11]. In addition, both DLCO and TLC showed a moderately strong negative correlation with the duration of ventilation (r = -0.43; p = 0.008 and r = -0.42; p = 0.01, respectively).

In a meta-analysis on 380 post-COVID-19 patients, an altered DLCO was reported in 39% of the overall population and in 66% of patients with severe illness [12]. Similar results were reported in a retrospective study by Huang *et al.* [13] on 57 COVID-19 patients. At 30-day follow-up, residual CT scan abnormalities were documented in 94.1% of severe and in 37.5% of nonsevere patients, with impaired DLCO being reported in more than 50% of the study population. In addition, a higher incidence of DLCO impairment (76.5 vs 42.5%) and a significantly lower percentage of predicted TLC and 6 min walking distance were reported in severe as compared with nonsevere patients.

In another study of 55 noncritical COVID-19 survivors evaluated 3 months after discharge, radiological and pulmonary abnormalities were observed in 25%. A reduced DLCO was the most frequent pulmonary abnormality, being reported in 16% of patients. In addition, the authors found that a higher value of D-dimer at admission was predictive of an impaired DLCO 3 months after discharge [14].

A large nationwide Swiss study recently investigated the pulmonary sequelae of COVID-19, 4 months after the onset of symptoms. Impairments in pulmonary function and physical performance were reported to be more pronounced in patients with severe and critical COVID-19 compared to those with mild and moderate disease. Thus, DLCO was particularly reduced in severe/critical COVID-19 patients, being related to a reduced walking distance and to exercise oxygen desaturation in the postacute phase. Moreover, a negative correlation between the duration of ventilation and both DLCO and TLC abnormalities was observed in mechanically ventilated patients during hospitalization [15].

The direct association between disease severity and the functional sequelae of COVID-19 was not confirmed when patients were evaluated at an earlier stage (30 days from symptoms onset). In particular, a recent study documented that DLCO, FVC and TLC (% predicted values) were not significantly different between groups of clinical and radiological severity, although significantly impaired in the overall population [16].

Overall, based on the results of the aforementioned studies, DLCO may be identified as a useful functional biomarker for COVID-19 patients upon discharge from acute care hospitals and admission to postacute care facilities (nursing homes, rehabilitation centers, home health agencies, etc.). DLCO reflects the gas-exchanging function of the alveolar–capillary barrier of the lungs, resulting from the product of the carbon monoxide transfer co-efficient (KCO) multiplied by alveolar volume (VA) [17]. KCO reflects the gas exchange per unit of lung volume and mainly depends on the thickness and area of the alveolar capillary membrane, the blood volume in capillaries supplying ventilated alveoli and hemoglobin concentration in the alveolar capillary blood. A decrease in DLCO may be due to a decrease in KCO, VA or both. Thus, it may be difficult to interpret which is the predominant mechanism of an impaired DLCO [17]. The pathological changes observed in the lungs of deceased COVID-19 patients can explain to a certain extent the DLCO impairment, since a characteristic of pulmonary SARS-CoV-2 infection is the extensive alveolar epithelial and endothelial cell injury, followed by secondary fibroproliferation [18].

Of interest, in about 50% of COVID-19 patients with impaired DLCO, the DLCO/VA value is still within the normal range, as reported by Mo *et al.* [10]. DLCO/VA takes into account the differences in lung size, thus it is sometimes considered as a more accurate expression of the intrinsic gas exchange function of the lung. The observation that in post-COVID-19 patients DLCO may be impaired while DLCO/VA may not be, could indicate that the alteration of the diffusion membrane has a prominent role in causing lung dysfunction compared with the reduced VA [10].

In conclusion, if we also consider the tropism of SARS-CoV-2 for alveolar epithelial cells [19], the evidence of abnormal lung function tests in post-COVID-19 patients raises doubts and fears regarding the possible fibrotic evolution of the disease. This calls for the urgent need for specifically designed postacute care strategies to predict and manage COVID-19 sequelae in a timely manner. In this regard, DLCO has the potential to become a useful functional biomarker for post-COVID-19 patients, particularly for those admitted to postacute care facilities. A systematic functional assessment should be considered for all moderate–severe COVID-19 patients at the time of acute hospital discharge, and such a multidisciplinary approach could be provided by individualized rehabilitation programs. Lung function tests may be regarded as indispensable tools for monitoring functional impairment, planning rehabilitation, managing complications and preventing long-term outcomes.
